# Retraction: Redefining Health: Implication for Value-Based Healthcare Reform

**DOI:** 10.7759/cureus.r8

**Published:** 2017-02-22

**Authors:** Ikhwanuliman Putera

**Affiliations:** 1 Faculty of Medicine, University of Indonesia

This article has been retracted by the publisher as it was published accidentally without the consent of the Editor-in-Chief. This was due to an administrative error, as the article is still undergoing editorial review by the Cureus editorial team. The article will be republished in full when it has undergone complete editorial review and is determined to be ready for publication by the Editor-in-Chief.

